# DOLAMA study: Effectiveness, safety and pharmacoeconomic analysis of dual therapy with dolutegravir and lamivudine in virologically suppressed HIV-1 patients

**DOI:** 10.1097/MD.0000000000016813

**Published:** 2019-08-09

**Authors:** Carmen Hidalgo-Tenorio, Luis López Cortés, Alicia Gutiérrez, Jesús Santos, Mohamed Omar, Carmen Gálvez, Sergio Sequera, Samantha Eisabeth De Jesús, Franciso Téllez, Elisa Fernández, Coral García, Juan Pasquau

**Affiliations:** a Unit of Infectious Diseases, Virgen de las Nieves University Hospital, Granada; b Department of Infectious Diseases, Virgen del Rocio University Hospitals, Seville; c Unit of Infectious Diseases, Virgen de las Victoria University Hospital, Málaga; d Unit of Infectious Diseases, Hospital Complex of Jaen; e Unit of Infectious Diseases, Hospital Torrecárdenas Hospital, Almería; f Unit of Infectious Diseases, University Hospital Puerto Real, Cádiz; g Internal medicine Service, Hospital Poniente, Almería, Spain.

**Keywords:** DOLAMA, dolutegravir, dual therapy

## Abstract

Dolutegravir (DTG) has shown effectiveness in combination with rilpivirine in with experience of antiretroviral therapy (ART) and with 3TC in naïve patients (GEMINI trial). The main objectives of this real-life study were to analyze the effectiveness and safety of 3TC plus DTG in virologically suppressed HIV-1 patients and to conduct a pharmacoeconomic analysis.

We conducted an observational, retrospective and multicenter study of HIV+ patients pretreated for at least 6 months with ART that was then simplified to 3TC + DTG for any reason. We gathered data on viral loads (VLs) during exposure to the DT, calculating the rate with VL < 50 copies/mL at week 48, and on associated adverse effects.

The 177 HIV+ patients were collected, 77.4% male, with average age of 48.5 years and mean count of 252.2cell/μL CD4+ nadir lymphocytes; 96.6% had VL < 50 copies/mL and 674 cells/μL CD4+ lymphocytes. Median time since HIV diagnosis was 15 years, and median ART duration was 13 years, and 34.5% of patients were on mono- or dual-therapy before the switch. At week 48, 82.4% of patients had VL < 50 cop/μL using an intention-to-treat (ITT) analysis, 89.6% according to mITT, and 96.7% according to Per-Protocol analysis. 3.3% patients had virological failure (VF). These effectiveness data and costs were compared with those for 2 reference triple therapies (DTG/ABC/3TC and EVG/cobi/FTC/TAF) in a cost minimization analysis, showing cost savings with administration of DTG+3TC (2741 €/year vs DTG/ABC/3TC and 4164 €/year vs EVG/cobi/FTC/TAF) and in a cost-effectiveness analysis, finding the DT to be the most cost-effective approach (ICER = −548 vs DTG/ABC/3TC and ICER = −4,627€ vs EVG/cobi/FTC/TAF)

The combination of 3TC with DTG appears to be a safe and effective option for the simplification of ART in pretreated and virologically stable HIV-positive patients, being cost-effective and offering the same effectiveness as the triple therapy it replaces.

## 1 Introduction

The life expectancy of HIV positive patients is currently very similar to that of the general population.^[1]^ Clinical practice guidelines recommend the initiation of ART in all patients diagnosed with HIV, regardless of their immune status,^[2]^ using a combination of 2 nucleoside analogue reverse transcriptase inhibitors (NRTIs) along with a third drug from the antiretroviral (ARV) family, such as a non-nucleoside reverse transcriptase inhibitor (NNRTI), boosted protease inhibitor (bPI) or integrase inhibitor (INI). Given the need to maintain ART for life to prevent disease progression and reduce the risk of morbidity and mortality, researchers have studied simplification options with new ARVs to minimize the toxicity and support the sustained administration and effectiveness of the treatment.^[3]^

Simplification strategies include dual therapies (DTs) with lamivudine (3TC), which have demonstrated their effectiveness in clinical trials in combination with bPI (OLE, SALT, ATLAS, DUAL)^[4–7]^ as well as with raltegravir^[8]^ and dolutegravir (DTG),^[9]^ proving effective in >95% of patients, although sample sizes have been small. Recently, 2 clinical trials of DT with DTG plus rilpivirine (SWORD 1 and 2) demonstrated a similar effectiveness to that of triple therapy with no increase in the risk of resistance and an improvement in bone and renal parameters.^[10]^ The combination of DTG with 3TC also yielded excellent results in naïve patients in the PADDLE^[11]^ and GEMINI^[12]^ trials.

The objective of this study was to analyze the effectiveness and safety of 3TC (300 mg/QD) plus DTG (50 mg/QD) at week 48 in a large sample of real-life in virologically suppressed HIV-1 patients and to conduct a pharmacoeconomic study of this combination. Secondary objectives were to establish:

(1) the frequency of adverse events;
(2) the impact of the DT on lymphocyte subpopulations and biochemical parameters; and
(3) the influence of the 184 V mutation on the effectiveness of the DT.

## 2 Patients and methods

### 2.1 Study design and setting

We conducted a multicenter, observational, and retrospective study of HIV patients pretreated for at least 6 months with virological suppression and no history of virological failure (VF) or presence of genotypic mutations that might compromise the effectiveness of either drug under study. DT was prescribed by attending physicians after obtaining the informed consent of the patients. The study included patients under outpatient treatment from seven Departments of Infectious Diseases in the regional health service who had initiated treatment with DTG (50 mg/QD) plus 3TC (300/QD) for any reason before 30 June 2017. The study was approved by the ethics committee of the hospital and registered with the Spanish Medicines Agency. The data were treated in compliance with Spanish legislation on personal data protection (organic law 15/1999, 13 December).

### 2.2 Treatment description

This was an observational retrospective study on prescriptions of approved drugs for the routine treatment of these patients and involved no direct pharmacological intervention

### 2.3 Inclusion criteria

Presence of HIV infection, age ≥18 years, receipt of suppressor ART, and plasma VL < 50 copies/mL and ≤1 blip for at least 6 months before study enrolment.

### 2.4 Exclusion criteria

The presence of active AIDS during the study period; co-infection with hepatitis B virus (HBV) treated with tenofovir (infection with HBV under effective treatment with 3TC and entecavir was not an exclusion criterion), pregnancy, previous presence of HIV genotyped with 3TC or DTG resistance mutations, and a history of adverse reactions to either drug.

### 2.5 Variables/data sources

Patients were followed for 48 weeks. At the baseline visit (V0), data were gathered on age, sex, active co-infections, toxic habits (tobacco, alcohol), risky practices, height, weight, bone, and/or renal toxicity from previous ART (osteopenia, osteoporosis, dyslipidaemia, diarrhoea, hepatotoxicity, etc), date of HIV diagnosis, VL and CD4 and CD8 lymphocyte counts at diagnosis, CD4 nadir, previous ART lines, reason for change to DT (toxicity, intolerance, simplification, optimization, and/or inconvenience, among others), and analytical data in the 3 months before the change to DT. Data were also gathered at V0 and at the end of the follow-up on: VL, CD4 and CD8 lymphocyte counts and percentages, CD4/CD8 ratio, total cholesterol, HDL, LDL, and triglycerides; and GOT, GPT, GGT, bilirubin, alkaline phosphatase, and glomerular filtration (CKD-EPI).

Clinical and analytical data and any adverse effects were recorded in follow-up visits at 6 ± 2 weeks (V1), 24 ± 8 weeks (V2) and 48 ± 8 weeks (V3) after the change.

The effectiveness of this DT (DTG + 3TC) was determined by calculating the proportion of participants achieving viral suppression (defined as plasma HIV RNA < 50 cop/mL) at week 48 according to intention-to treat (ITT),^[13]^ modified intention-to treat (mITT) ^[14]^ and per-protocol (PP) analyses.^[15]^ The following patients were excluded from mITT analysis: those with a life expectancy of <1 year, those asking to return to the previous ART in STR solely because they preferred a single pill, and those for whom the DT was started but then changed because they were prescribed with a drug incompatible with DTG plus 3TC.

A blip was defined by VL > 50 HIV-RNA copies/μL at 1 determination and VL < 50 HIV-RNA copies at the next

VF was defined by VL > 50 HIV-RNA copies/μL at 2 consecutive determinations.

### 2.6 Study size

A sample size of at least 151 patients was estimated to achieve an accuracy of 5% in the estimation of a proportion by means of a normal 95% CI, assuming that the proportion was 91% (effectiveness reported in the previous study) and assuming possible losses of 20%.

### 2.7 Statistical analysis

Means and standard deviations were calculated for quantitative variables, followed by the application of Student *t* test for independent variables or, for variables found to be non-normally distributed, the Mann–Whitney *U* test. Absolute and relative frequencies were calculated for qualitative variables followed by application of Pearson chi-square test or, when conditions were not met, Fisher chi-square test. The Kolmogorov–Smirnov test was applied to check the normality of variable distributions. SPSS 20.0 was used for statistical analyses.

### 2.8 Pharmacoeconomic analysis

In the pharmacoeconomic analysis, the DTG + 3TC regimen was compared with DTG/ABC/3TC and EVG/cobi/FTC/TAF as widely used reference treatments. Effectiveness data based on an intention-to-treat (ITT) analysis were obtained for DTG + 3TC from the present study, for DTG/ABC/3TC from the STRIIVING trial,^[16]^ and for EVG/cobi/FTC/TAF from the STUDY 109 trial,^[17]^ which are both clinical trials in patients with a history of ART, since it would not be methodologically correct to carry out this analysis using data from trials conducted under different conditions.

We calculated the annual costs of each strategy according to the list of retail laboratory prices consulted in September 2018, without adding 4% VAT or subtracting the 7.5% discount agreed with the government to reduce the public deficit. It is usual in this type of research to study the gross price of the drug without considering value-added tax or administration discounts in order to facilitate comparisons of the price per unit of effectiveness among different countries. No account was taken of the costs of managing possible adverse effects, changing strategies or conducting resistance studies.

We conducted a cost-minimization analysis to estimate the saving that would result from the adoption of DT with DTG+3TC instead of the triple therapies. We also carried out a cost-effectiveness ratio analysis, dividing the cost of each treatment by its effectiveness for virological suppression. The incremental cost-effectiveness ratio was determined by dividing the cost differences by the effectiveness increase.

## 3 Results

### 3.1 Study population

The study included 177 HIV+ patients, 77.4% males, mean age of 48.5 years, mean CD4+ lymphocyte nadir of 252.2 cells/μL, diagnosis of HIV for a median of 15 years (IQR: 7–22), median of 13 years under ART (IQR: 4–18) and median of 5 previous treatment lines (IQR: 2–8); 96.6% had baseline VL of < 50 copies/mL and mean CD4 lymphocyte count of 697.7cells/μL; 38.9% had previously received INI. Results for the remaining study variables are exhibited in Table 1.

**Table 1:**
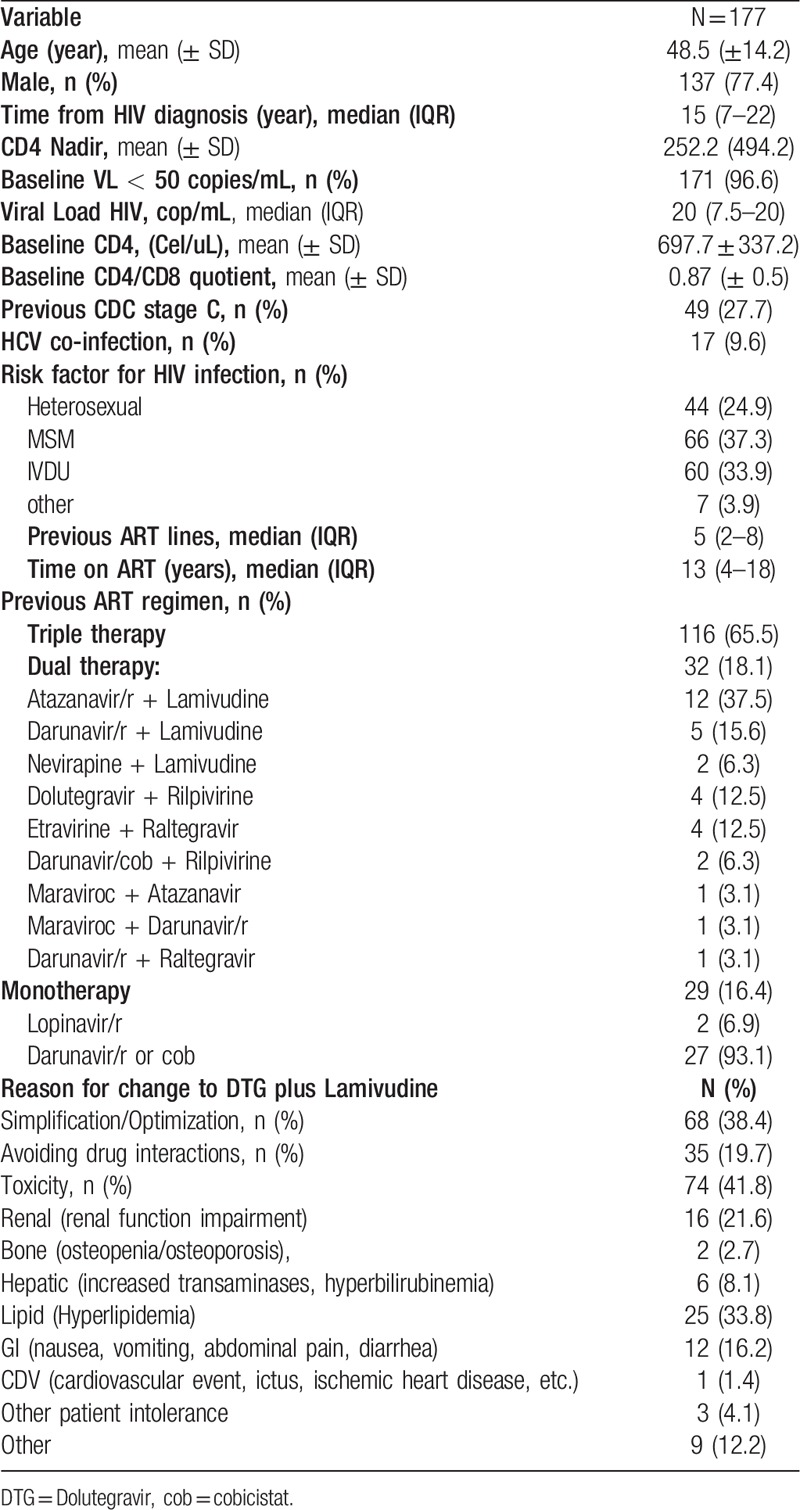
General description of the population.

**Figure 1:**
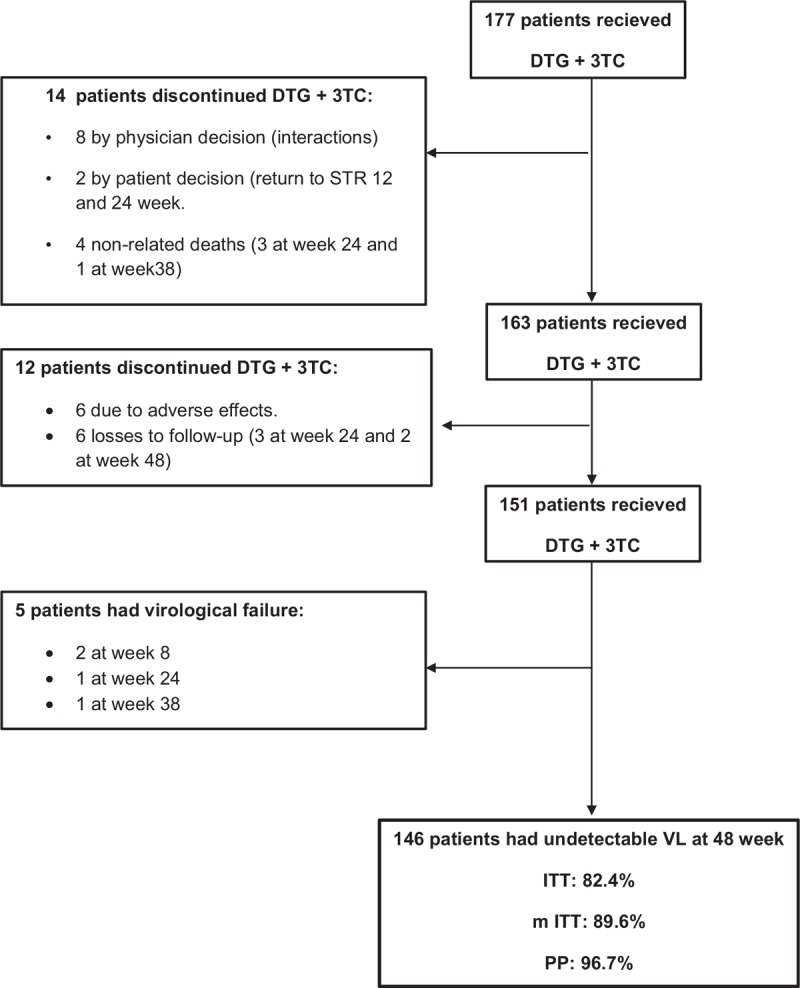
Flowchart of patients with Dolutegravir plus Lamivudine.

The main reasons for changing to DT with DTG and 3TC were toxicity of the previous regimen (41.8%), simplification/optimization (38.4%) and avoidance of drug interactions (19.7%) (Table 1). In patients receiving monotherapy, the change to this DT was due to toxicity (hyperlipemia, abdominal pain, ischemic heart disease) in 22 (75.9%) of the patients and drug interactions in 7 (24.1%). In patients already receiving DT, the change was due to adverse events (hyperbilirubinemia, hyperlipidemia, hematuria/crystalluria, gastrointestinal discomfort/digestive intolerance, renal toxicity) in 15 (46.9%), drug interactions in 9 (28.1%) and simplification (reduction in n° tablets and/or doses) in 8 (25%). Figure 1 depicts the flow of patients. Among the 177 enrolled patients, 26 (14.7%) dropped out of the study for: adverse events (n = 6); physician decision (n = 8: to start hepatitis C virus [HCV] treatment with direct-acting antivirals in 5 cases, to change to triple-therapy with Abacavir due to poor compliance in 1 case, and due to drug interaction in 2 cases); patient decision to return to a single pill (n = 2: weeks 12 and 24); loss to follow-up (n = 6); and non-related death (n = 4: lung adenocarcinoma stage IV, terminal esophagus carcinoma, decompensated liver cirrhosis, and sudden death due to severe atherosclerosis with stenosed vessels).

### 3.2 DT effectiveness

At week 48, DTG plus 3TC was effective in 96.7% of patients according to per-protocol (PP) analysis, 89.6% using mITT analysis and 83.6% using ITT analysis. Among the 151 patients in the final study sample, 5 (3.3%) had VF (2 at week 8, 1 at week 24, and 1 at week 38).

Two of the 5 patients with VF underwent a genotypic resistance test, showing no amplification in one and detecting K103R and S147G mutations in the other (previously exposed to ABC/3TC + RAL); neither mutation reduces susceptibility or produces resistance to INI. The three remaining patients did not undergo a resistance study (Table 2).

**Table 2:**
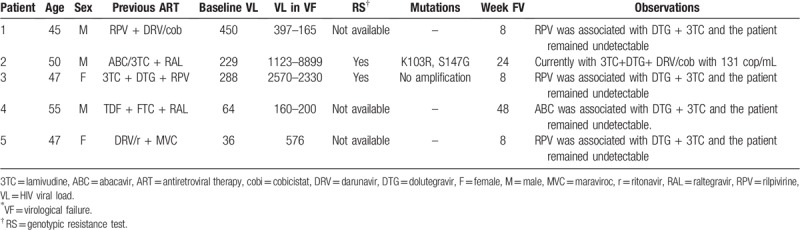
Virological failures.

Among the 178 patients, 90 (50.5%) had undergone a baseline genotypic resistance test. Among the four patients (4.4%) with 184 V mutation, VF was recorded in 1 (25%) but not in the other 3 (*P* = .129).

### 3.3 Analytical parameters

The change to DT resulted in a significant (*P* = .023) increase in the CD4/CD8 ratio, significant reductions of 8 mg/dL in total cholesterol (*P* = .002), 25 mg/dL in HDL (*P* = .002), and 48 mg/dL in triglycerides (*P* = .0001), and a significant increase of 14 mg/dL in LDL (*P* = .003) and in the total cholesterol/HDL ratio (*P* = .0018) (Table 3). These results were not affected by previous ART with tenofovir or abacavir (Table 4). We observed a decrease in liver enzyme levels and a small increase in creatinine levels, but these changes were not clinically relevant (Table 3).

**Table 3:**
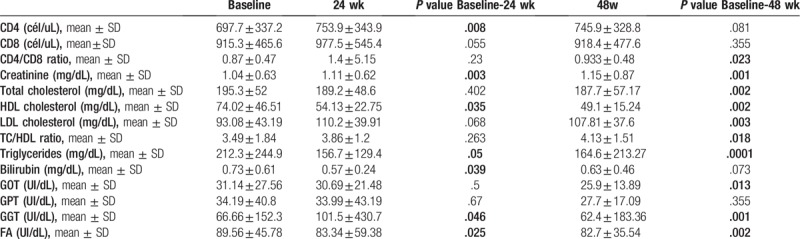
Analytical changes between baseline visit and week 24, 48 of all patients.

**Table 4:**
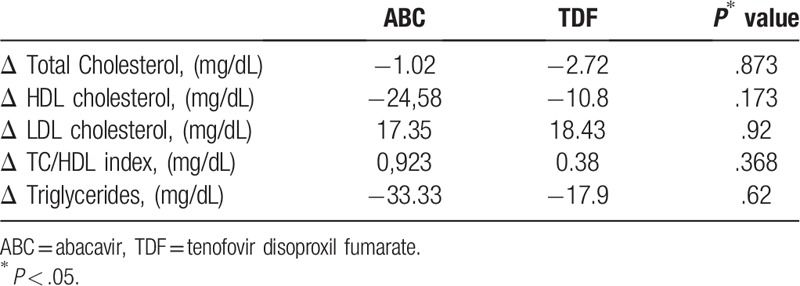
Lipid profile change according to history of treatment with ABC or TDF at week 48 of all patients.

### 3.4 Adverse effects

The adverse effects leading to the discontinuation of DTG + 3TC treatment in 6 patients were: grade 2 diarrhea in week 2 (n = 1); insomnia and anxiety in week 4 (n = 2); asthenia in week 11 (n = 1); insomnia in week 13 (n = 1); and hypercholesterolemia in week 18 (n = 1).

### 3.5 Pharmacoeconomic analysis

The annual cost of treatment was estimated to be 7759 € for DTG + 3TC, compared with 10,500 € for DTG/ABC/3TC and 11,923 € for EVG/cobi/FTC/TAF. Change to this DT would therefore generate an annual saving of 2741 € (26%) vs DTG/ABC/3TC and 4164 € (35%) vs EVG/cobi/FTC/TAF. Virological effectiveness rates were 90, 83, and 97% for DTG + 3TC, DTG/ABC/3TC and EVG/cobi/FTC/TAF, respectively (Table 5). Cost-effectiveness ratios were 86, 127, and 123, respectively, making DTG + 3TC the more cost-effective option for HIV treatment. Incremental cost-effectiveness ratios were “−392” for DTG/ABC/3TC and “+595” for EVG/cobi/FTC/TAF, indicating the slightly higher virological effectiveness in comparison to DTG/ABC/3TC and the lower cost of the DT in comparison to these reference therapies (Table 5).

**Table 5:**
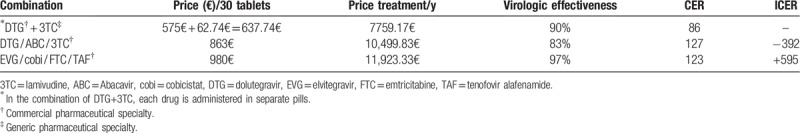
Cost of ART and virologic effectiveness. Results of CER and ICER analyses.

## 4 Discussion

The dual therapy with DTG plus 3TC proved to be a safe and effective treatment in this population of mainly middle-aged virologically suppressed individuals with a long history of HIV infection, extensive exposure to ART (8 or more ARV lines in around a quarter of cases) and even with previous experience of INIs. Among the patients who completed the follow-up at week 48, DT achieved an undetectability rate close to 100%. Similar results have been reported for DT with DTG plus 3TC. Thus, the LAMIDOL study found that 97.1% of 110 patients with previous ART had undetectable VL at week 48 of treatment,^[18]^ and another investigation reported that 97% of 72 pretreated patients had undetectable VL at week 96.^[19]^ Comparable results for DTG plus 3TC have also been published by an Italian group,^[20]^ by the DOLULAM study, in which one-third of patients had the 184 V mutation,^[21]^ and by the ACTG A5353 study.^[22]^ In a retrospective study that compared DTG plus 3TC with 3TC plus boosted PIs in virologically suppressed HIV-1-positive individuals found that a switch to 3TC and DTG yielded similar efficacy but longer durability.^[23]^

In the present investigation, the presence of the 184 V mutation did not compromise the virological effectiveness of the DT, as also reported by the DOLULAM study.^[21]^ There were 5 cases of VF in our study population (3.3%), and resistance analysis in one of these patients showed no resistance mutations to either ARV. Four of the five patients achieved virological suppression after the association of a third ARV, whereas VF persisted in the remaining patient despite triple therapy, attributed to poor adherence to the treatment. Likewise, another retrospective study found that the presence of the 184 V resistance mutation alone was not a predictor of VF, although they observed that peak HIV-RNA > 5,000,000 copies/mL independently predicted VF after adjusting for the presence of the M184 V resistance mutation and duration of virological suppression.^[24]^ In addition, a recent clinical trial of 91 pretreated patients randomized into 3 branches (triple therapy, DT (DTG + 3TC), and DTG in monotherapy) reported that 1 mutation-free VF developed in the DT branch.^[25]^ However, no case of VF was observed in a trial of 203 pretreated HIV-positive patients who received DTG+3TC, with a follow-up of 295 patient/years.^[20]^ Finally, the ASPIRE trial found DTG + 3TC and triple therapy to be equally effective in pretreated patients at weeks 24 and 48.^[26]^

The CD4/CD8 ratio was improved by this DT. It naturally decreases with age, is associated with increased mortality, and is a marker of both acute and chronic inflammation; after the initiation of ART, there is an increase in CD4^+^ cell count and decrease in CD8^+^ cell count. These data suggest that an increasing CD4:CD8 ratio alongside undetectable HIV VL could be markers of effective ARV treatment with DT and a good prognosis.^[27]^

There were changes in the lipid profile, with an increase in CT/HDL ratio regardless of previous treatment with tenofovir or abacavir. Therefore, this DT seems to have a more atherogenic profile in comparison with previous ART.

There was a small increase in creatinine levels. DTG inhibits organic cation transporter 2 on the basolateral side of proximal tubule cells of the kidney and leads to increased serum creatinine levels but no true impairment of renal function.^[28]^

The tolerance was good, with only 3.4% discontinuing the DT regimen due to an adverse effect (insomnia in 1.7% of patients), always during the first 3 months of treatment. Very similar findings were recently described for raltegravir and DTG in a triple-therapy regimen, with 3.6% discontinuing DTG for toxic effects, which were neurological in 1.7% of the patients and associated with the female sex; they also reported a VF rate of 0.1%.^[29]^

Our comparative cost-effectiveness study was based on the present data and the available scientific evidence in pretreated HIV infected patients. Our results are similar to those of other studies on DT with DTG plus 3TC in pretreated HIV infected patients.^[20,30]^ Although the results do not rule out DTG/ABC/3TC or EVG/cobi/FTC/TAF as cost-effective options,^[31]^ especially in patients who are not good candidates for DT, the combination of DTG and 3TC proved to be a more cost-effective approach, reducing the costs by patient/yrs.

Limitations of our study include its retrospective, open-label and non-randomized design. The restrictive exclusion criteria also limited information on the effectiveness and safety of DT in the excluded population. In addition, genotypic resistance testing was performed in only 2 of the 5 patients who developed VF, preventing conclusions being drawn on the reason for the failure of this DT. One of the main strengths of this multicenter investigation is the large sample size of patients in a real-life clinical setting rather than a clinical trial

In conclusion, these results suggest that the combination of 3TC and DTG is a novel, effective, safe and cost-effective option for ART simplification in virologically stable pre-treated patients without 3TC or DTG resistance mutations, even in those with previous experience of INIs. It appears to be no less effective than the triple therapy it replaces.

## Author contributions

**Conceptualization:** Carmen Hidalgo Tenorio, Jesús Santos, Coral García, Juan Pasquau.

**Data curation:** Alicia Gutiérrez, Jesús Santos, Mohamed Omar, Carmen Galvez, Sergio Sequera, Samantha Eisabeth De Jesus, Franciso Tellez, Elisa Fernandez, Coral García, Juan Pasquau.

**Formal analysis:** Carmen Hidalgo Tenorio, Sergio Sequera, Samantha Eisabeth De Jesus, Juan Pasquau.

**Investigation:** Carmen Hidalgo Tenorio, Samantha Eisabeth De Jesus, Coral García, Juan Pasquau.

**Methodology:** Carmen Hidalgo Tenorio, Samantha Eisabeth De Jesus, Coral García, Juan Pasquau.

**Project administration:** Carmen Hidalgo Tenorio, Samantha Eisabeth De Jesus, Coral García.

**Resources:** Mohamed Omar, Carmen Galvez, Coral García.

**Software:** Alicia Gutiérrez, Jesús Santos, Mohamed Omar, Carmen Galvez, Sergio Sequera, Samantha Eisabeth De Jesus, Franciso Tellez, Elisa Fernandez, Coral García.

**Supervision:** Luis López Cortés, Carmen Galvez, Sergio Sequera, Elisa Fernandez, Juan Pasquau.

**Validation:** Luis López Cortés, Carmen Galvez, Sergio Sequera, Franciso Tellez, Coral García, Juan Pasquau.

**Visualization:** Jesús Santos, Carmen Galvez, Sergio Sequera, Juan Pasquau.

**Writing – original draft:** Carmen Hidalgo Tenorio.

**Writing – review & editing:** Sergio Sequera, Juan Pasquau.
